# Brazilian network for HIV Drug Resistance Surveillance (HIV‐BresNet): a survey of treatment‐naive individuals

**DOI:** 10.1002/jia2.25032

**Published:** 2018-03-05

**Authors:** Monica B Arruda, Lídia T Boullosa, Cynthia C Cardoso, Carolina M da Costa, Carlos Brites, Shirlene TS de Lima, Helena T Kaminski, Agdemir W Aleixo, Ana OP Esposito, Ana MS Cavalcanti, Maristela Riedel, José C Couto‐Fernandez, Selma B Ferreira, Ivi CM de Oliveira, Loreci E Portal, Hilda HC Wolf, Sandra B Fernandes, Maria I de M. C. Pardini, Manoel VC Feiteiro, Fernanda M Tolentino, Ricardo S Diaz, Giselle ISL Lopes, Roberta BL Francisco, Nazle MC Véras, Ana F Pires, Miriam Franchini, Fábio Mesquita, Amilcar Tanuri, HIV‐BResNet, Andrea de Melo Xavier Shimizu, Célia Regina Mayoral Pedroso Jorge, Leda Maria Simões Mello, Eider Gurgel de Freitas, Unaí Tupinambás, Dijane Cristina de Barros Rosa Costa, Sirleide Pereira da Silva, Maria da Graça Winhescki, Carlos Silva de Jesus, Érica Ramos dos Santos Nascimento, Fatima Ercília de Oliveira Prazim, Marilda Tereza Mar da Rosa, Karina Salvador, Senele Ana de Alcântara Belettini, Rejane Maria Tommasini Grotto, Paulo Henrique de Oliveira, Érica Valessa Ramos Gomes, Danilo Araujo Dias, Juliana Galinskas, Norberto Camilo Campos, José Boullosa Alonso Neto

**Affiliations:** ^1^ Laboratório de Virologia Molecular Departamento de Genética‐IB Universidade Federal do Rio de Janeiro Rio de Janeiro RJ Brazil; ^2^ Fundação de Medicina Tropical do Amazonas Manaus AM Brazil; ^3^ Laboratório de Pesquisa LAPI Universidade Federal da Bahia Hospital Universitário “Prof. Edgar Santos” Salvador BA Brazil; ^4^ Laboratório Central de Saúde Pública do Ceará (Lacen‐CE) Fortaleza CE Brazil; ^5^ Laboratório Central de Saúde Pública do Distrito Federal Setor de Grandes Areas Norte (SGAN) 601 Brasilia DF Brazil; ^6^ Faculdade de Medicina Laboratório de Imunologia e Biologia Molecular (DIP) Universidade Federal de Minas Gerais (UFMG) Belo Horizonte MG Brazil; ^7^ Laboratório Central de Saúde Pública de Mato Grosso do Sul Campo Grande MS Brazil; ^8^ Laboratório Central de Saúde Pública de Pernambuco Recife PE Brazil; ^9^ Laboratório Municipal de Curitiba Curitiba PR Brazil; ^10^ Laboratório de AIDS e Imunologia Molecular Departamento de Imunologia FIOCRUZ Rio de Janeiro RJ Brazil; ^11^ UFRJ, Laboratório de Carga Viral Hospital Universitário Clementino Fraga Filho Rio de Janeiro RJ Brazil; ^12^ Instituto de Biologia do Exército Rio de Janeiro RJ Brazil; ^13^ Laboratório Central de Saúde Pública do Rio Grande do Sul Porto Algre RS Brazil; ^14^ Laboratório do Hospital Nossa Senhora da Conceição Porto Alegre RS Brazil; ^15^ Laboratório Central de Saúde Pública de Santa Catarina Florianópolis SC Brazil; ^16^ Laboratório de Biologia Molecular do Hemocentro de Botucatu Faculdade de Medicina UNESP Botucatu SP Brazil; ^17^ Laboratório de Pesquisa em AIDS‐Hospital de Clíncas da UNICAMP Campinas SP Brazil; ^18^ Laboratório de Biologia Molecular‐Instituto Adolfo Lutz de São José do Rio Preto São José do Rio Preto SP Brazil; ^19^ Escola Paulista de Medicina Laboratório de Retrovirologia Universidade Federal de São Paulo (UNIFESP) São Paulo SP Brazil; ^20^ Laboratório de Retrovírus Núcleo de Doenças Sanguíneas e Sexuais Centro de Virologia Instituto Adolfo Lutz Central São Paulo SP Brazil; ^21^ Departamento de Vigilância Prevenção e Controle das DST, AIDS e Hepatites Setor Administrativo Federal Sul (SAFS) 02 Secretaria de Vigilância em Saúde Ministério da Saúde Brasília DF Brazil; ^22^ Programa de Pós Graduação em Saúde Coletiva Faculdade de Medicina Faculdade de Ciências de Saúde Universidade de Brasília Brasília DF Brazil; ^23^ Faculdade de Medicina Universidade de São Paulo São Paulo SP Brazil

**Keywords:** HIV, HIV drug resistance, pretreatment HIV drug resistance, primary antiretroviral resistance, antiretroviral resistance, HIV Drug Resistance Surveillance

## Abstract

**Introduction:**

In Brazil, more than 487,450 individuals are currently undergoing antiretroviral treatment. In order to monitor the transmission of drug‐resistant strains and HIV subtype distribution in the country, this work aimed to estimate its prevalence and to characterize the nationwide pretreatment drug resistance in individuals recently diagnosed with HIV between 2013 and 2015.

**Methods:**

The HIV threshold survey methodology (HIV‐THS, WHO) targeting antiretroviral‐naive individuals with recent HIV diagnosis was utilized, and subjects were selected from 51 highly populated cities in all five Brazilian macroregions. The HIV *pol* genotypic test was performed by genomic sequencing.

**Results:**

We analysed samples from 1568 antiretroviral‐naive individuals recently diagnosed with HIV, and the overall transmitted drug resistance (TDR) prevalence was 9.5% (150 sequences). The regional prevalence of resistance according to Brazilian geographical regions was 9.4% in the northeast, 11.2% in the southeast, 6.8% in the central region, 10.2% in the north and 8.8% in the south. The inhibitor‐specific TDR prevalence was 3.6% for nucleoside reverse transcriptase inhibitors (NRTIs), 5.8% for non‐nucleoside reverse transcriptase inhibitors (NNRTIs) and 1.6% for protease inhibitors (PIs); 1.0% of individuals presented resistance to more than one class of inhibitors. Overall, subtype B was more prevalent in every region except for the southern, where subtype C prevails.

**Conclusions:**

To the best of our knowledge, this is the first TDR study conducted in Brazil with nationwide representative sampling. The TDR prevalence revealed a moderate rate in the five Brazilian geographical regions, although some cities presented higher TDR prevalence rates, reaching 14% in São Paulo, for example. These results further illustrate the importance of surveillance studies for designing future strategies in primary antiretroviral therapy, aiming to mitigate TDR, as well as for predicting future trends in other regions of the globe where mass antiretroviral (ARV) treatment was implemented.

## 1 Introduction

The Brazilian Ministry of Health implemented, in 1996, a pioneering programme for the care and support of people living with HIV/AIDS, which encompasses free universal access to antiretroviral drugs for HIV‐infected individuals. Besides the undoubted benefits of such policy, antiretroviral resistance remains one of the major obstacles to sustain HIV suppression during antiretroviral therapy (ART) 1, 2. Transmitted drug resistance (TDR), has been associated with first‐line antiretroviral virological failure in Brazil 3, and may as well compromise other therapeutic interventions, such as pre‐exposure prophylaxis (PrEP), prevention of mother‐to‐child transmission (PMTCT) and post‐exposed prophylaxis (PEP) 2.

It is important to highlight that the treatment practices adopted by different countries can greatly influence TDR rates and substantial differences can be reported worldwide. In high‐income countries, the number of newly infected patients that carry at least one major drug resistance mutation can vary from 7% to 17% 2, 4, 5. A recent study analysed samples from 26 European countries and reported an overall prevalence of TDR of 8.3%. Countrywide studies identified TDR rates of 11.2% in the United States 6, 5.6% in Sweden 7, 14.7% in Romania 8 and 9.9% in Spain 9. In middle‐ and low‐income countries, the prevalence of TDR is around 7.0%, estimated at 6.3% in Latin America 4, 5.7% in India 10 and less than 5.0% in major African countries, including Angola, Botswana, South Africa, Uganda, Zimbabwe and Chad 11.

Brazil has recently adopted the Test and Treat policy and has an increasing number of patients in antiretroviral treatment at specialized AIDS clinics. In December 2015, more than 455,000 individuals were receiving antiretroviral treatment in Brazil, accounting for almost 68% of the HIV‐infected individuals who are followed by the Brazilian Public Health System.

In order to monitor the transmission of drug‐resistant strains, as well as the HIV subtype distribution in Brazil, the Brazilian Ministry of Health has established the National Network for Drug Resistance Surveillance (HIV‐BresNet). The first survey, conducted on 2001, showed an overall rate of transmitted resistance of 6.6%, with an even distribution of protease and reverse transcriptase inhibitors resistance‐related mutations 12. In the second survey, carried out between 2007 and 2008, TDR mutations were found in 8.1% of the studied population, and an intermediate level of transmitted resistance (between 5% and 15%) was found in major Brazilian cities, such as Belem, Brasilia, São Paulo and Rio de Janeiro 13.

Brazil is a continental country, and its different geographical regions have unique demographics as well as AIDS incidence. Therefore, determining a homogeneous picture of the Brazilian HIV/AIDS epidemic may be challenging. In order to provide important data for the elaboration and revision of policies regarding prevention, treatment and care of HIV, this work aimed to estimate its prevalence and characterize the nationwide pretreatment drug resistance in individuals recently diagnosed with HIV, referred to as the first viral load test.

## 2 Methods

### 2.1 Sampling

A countrywide sampling was conducted in Brazil's five major geographical regions: north, northeast, central‐west, southeast and south. For this purpose, we have included 72 laboratories from the Brazilian Network for HIV Viral Load testing in 51 cities throughout the country. All patients were recently diagnosed and samples were collected before ARV onset. Each region had probability proportional to size (PPS) sampling based on the information of the number of people who initiated ART in the previous time period. The standard sample size was 254, according to the WHO protocol for Surveillance of HIV drug resistance in adults initiating ART 14. Based on it, at least 254 samples were collected per region and distributed for each state maintaining the ratio of samples tested for viral load for the first time between 2013 and 2015. In view of the HIV/AIDS epidemic in Brazil, which is highly concentrated in the southeast, the sample size was doubled to 508 in this region to prevent any sampling bias. The criteria for inclusion were: (i) 18 years old or older; (ii) first viral load at the Brazilian Ministry of Health National Network Laboratories; and (iii) ART‐naive individuals according to the Brazilian National System of Drugs Logistic Control (SICLOM). The ethical issues of this study were reviewed by UFRJ‐IRB under # 30459614.9.0000.5257. This study was approved without the need for signed consent from volunteers, allowing the use of information available on the application form used for viral load exams only.

### 2.2 Genotypic analyses

Nineteen laboratories were responsible for running the HIV genotypic test using the TRUGENE® HIV‐1 Genotyping Kit and the OpenGene® DNA Sequencing System (Siemens Healthcare Diagnostics, Tarrytown, NY, USA) and the quality of the test was assured using an external quality proficiency panel distributed by the Brazilian Ministry of Health, as previously described 15. Resistance mutations were assigned by the Calibrated Population Resistance (CPR) algorithm 16, which is based on the WHO drug resistance mutation list for surveillance of transmitted HIV‐1 drug resistance 17. Additional analyses were conducted in order to verify pretreatment drug resistance, following WHO guidelines 14, using the HIVdb Program 18. HIV‐1 subtype assignments were defined according to the REGA HIV‐1 Automated Subtyping Tool 19, 20 and the HIVdb Program 18.

### 2.3 Statistical analysis

Results of qualitative variables were represented as total counts and frequency and a Pearson's Chi‐squared test was applied for comparisons between genders, geographical regions and HIV subtypes. Results of quantitative variables (age and viral load) were represented as mean and standard deviation. Adherence to normal distribution was assessed using the Shapiro‐Wilk test. A Kruskal‐Wallis rank sum test was applied for overall comparisons of quantitative data between regions and a Dunn's *post hoc* test was applied for pairwise comparisons with the Bonferroni adjustment. Age and viral load comparisons between genders were performed using a Mann‐Whitney test. All analyses were performed using R for Windows 3.2.0 (R Development Core Team, Vienna, Austria).

## 3 Results

### 3.1 Sampling

In this study, samples were collected from October 2013 to January 2015, at the 72 viral load laboratories members of the Brazilian Ministry of Health National Network Laboratories. From this sampling, 1568 had the first 1000 nucleotides of *pol* region appropriately sequenced (GenBank accession numbers KX887502 to KX889067), attending the CPR algorithm. With the exception of the southeast region, which had 500 sequenced samples instead of the estimated number (508 samples), the sampling size inferred according to the PPS methodology was reached (see Supporting Information).

Information concerning demographic parameters such as age and gender, as well as viral loads, was available for 1319 individuals. In general, the number of male samples (N = 919, 70%) was higher than female (N = 400, 30%), with the former composed of significantly younger individuals when compared to the latter (*p* < 0.0001). The viral load was also significantly higher in males (4.79 ± 0.90 log10 copies/ml) than females (4.61 ± 0.90 log10 copies/ml) (*p* < 0.001) (Table 1). Comparisons of viral loads according to geographical region have also shown a statistically significant variation (*p* < 0.001; Kruskal‐Wallis test). Results of *post hoc* comparisons have showed that viral loads were significantly lower in the southeast (4.602 ± 0.876) as compared to the northeast (4.857 ± 0.927) and southern (4.835 ± 0.924) regions (*p* < 0.01).

**Table 1:** Distribution of age, gender and viral loads according to region^a^

	Total	North	Northeast	South	Southeast	Central‐West
Age (years)^b^	35 ± 12	34 ± 11	35 ± 11	37 ± 12	35 ± 12	36 ± 12
Male	34 ± 11.5	34 ± 11	34 ± 12	35 ± 12	34 ± 11	35 ± 11
Female	37 ± 12.5	34 ± 12	37 ± 10	38 ± 13	38 ± 13	37 ± 13
Gender^c^						
Male	919 (70)	168 (68)	119 (70)	153 (64)	336 (72)	143 (72)
Female	400 (30)	78 (32)	52 (30)	86 (36)	129 (28)	55 (28)
Viral load						
Male^d^	4.793 ± 0.901	4.8 ± 0.9	4.899 ± 0.889	4.942 ± 0.877	4.647 ± 0.879	4.872 ± 0.931
Female	4.614 ± 0.896	4.684 ± 0.846	4.762 ± 1.012	4.652 ± 0.977	4.483 ± 0.862	4.634 ± 0.768
Total^e^	4.739 ± 0.903	4.763 ± 0.898	4.857 ± 0.927	4.835 ± 0.924	4.602 ± 0.876	4.805 ± 0.892

a Data is presented as N (%) for gender and mean ± SD (standard deviation) for age and viral loads.

b *p* < 0.0001 for comparisons according to regions (Kruskal‐Wallis test).

c Total counts do not add up to the total number of subjects in this study (1319 against 1568 individuals) due to missing information.

d *p* < 0.001 for comparisons of viral loads according to gender in the total sample (All) and *p* < 0.05 for the same comparison in the south and southeast regions (Mann‐Whitney test).

e *p* < 0.001 for comparisons of viral loads according to regions (Kruskal‐Wallis test) and *p* < 0.01 for comparisons between southeast and northeast and between southeast and south regions (Dunn's *post hoc* test).

### 3.2 TDR analyses

TDR analyses based on the CPR algorithm were conducted for each geographical region separately (see Supporting Information for details). The presence of any TDR in the analysed sequences from each Brazilian region varied from 6.8% (n = 18) in the central‐west region to 11.2% (n = 56) in the southeast region. The prevalence of resistance to each drug class was similar in the different Brazilian regions (Table 2).

**Table 2:** Prevalence of drug resistance according to region

	All (N = 1568)^a^	North (N = 265)	Northeast (N = 265)	South (N = 273)^a^	Southeast (N = 500)	Central‐West (N = 265)
NRTI	57 (3.6;2.8 to 4.7)	13 (4.9; 2.7 to 8.4)	11 (4.1; 2.2 to 7.5)	5 (1.8; 0.7 to 4.5)	20 (4; 2.5 to 6.2)	8 (3; 1.4 to 6.1)
NNRTI	91 (5.8; 4.7 to 7.1)	14 (5.3; 3 to 8.9)	12 (4.5; 2.5 to 8)	19 (7; 4.3 to 10.8)	34 (6.8; 4.8 to 9.5)	12 (4.5; 2.5 to 8)
NRTI/NNRTI	131 (8.3; 7 to 10)	24 (9; 6 to 13.3)	21 (7.9; 5.1 to 12)	21 (7.7; 4.9 to 11.7)	49 (9.8; 7.4 to 12.8)	16 (6; 3.6 to 9.8)
PI^b^	25 (1.6; 1 to 2.4)	6 (2.2; 0.9 to 5.1)	6 (2.2; 0.9 to 5.1)	3 (1.1; 0.3 to 3.4)	9 (1.8; 0.9 to 3.5)	1 (0.4; 0.02 to 2.4)
Any resistance	150 (9.5; 8.2 to 11.1)	27 (10.2; 6.9 to 14.6)	25 (9.4; 6.3 to 13.8)	24 (8.8; 5.8 to 12.9)	56 (11.2; 8.6 to 14.4)	18 (6.8; 4.2 to 10.7)

Results are presented as N (%; 95% CI). NRTI, nucleoside reverse transcriptase inhibitor; NNRTI, non‐nucleoside reverse transcriptase inhibitor; PI, protease inhibitor.

a *p* < 0.01 for comparisons between prevalence of resistance to NNRTI and NRTIs.

b *p* < 0.01 for comparisons between prevalence of resistance to PIs and NRTI/NNRTI in the entire sample (All) and in each region separately.

**Figure 1:**
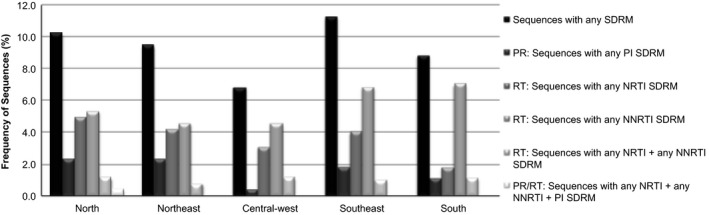
Prevalence of sequences with any SDRM (surveillance drug‐resistance mutation), any nucleoside reverse transcriptase inhibitors (NRTI) SDRM, any non‐nucleoside reverse transcriptase inhibitors (NNRTI) SDRM and protease inhibitors (PI) SDRM distributed throughout all five geographical regions in Brazil: north, northeast, central‐west, southeast, and south.

When considering each antiretroviral class a higher prevalence of TDR was identified for non‐nucleoside reverse transcriptase inhibitors (NNRTIs), ranging from 4.5% (n = 12) in the northeast and central‐west to 7.0% (n = 19) in the south (Table 2, Figure 1). Prevalence of TDR to NNRTIs was significantly higher than to nucleoside reverse transcriptase inhibitors (NRTIs) in an analysis considering subjects from all regions and also among subjects from the southern region (*p* < 0.01, both). As expected, prevalence of resistance to reverse transcriptase inhibitors (NRTI/NNRTI) was significantly higher than to protease inhibitors (PI; *p* < 0.01) in all Brazilian regions (Table 2). The north was the only region which showed one sequence with TDR to three antiretroviral classes (n = 1, 0.4%) (Figure 1).

**Figure 2:**
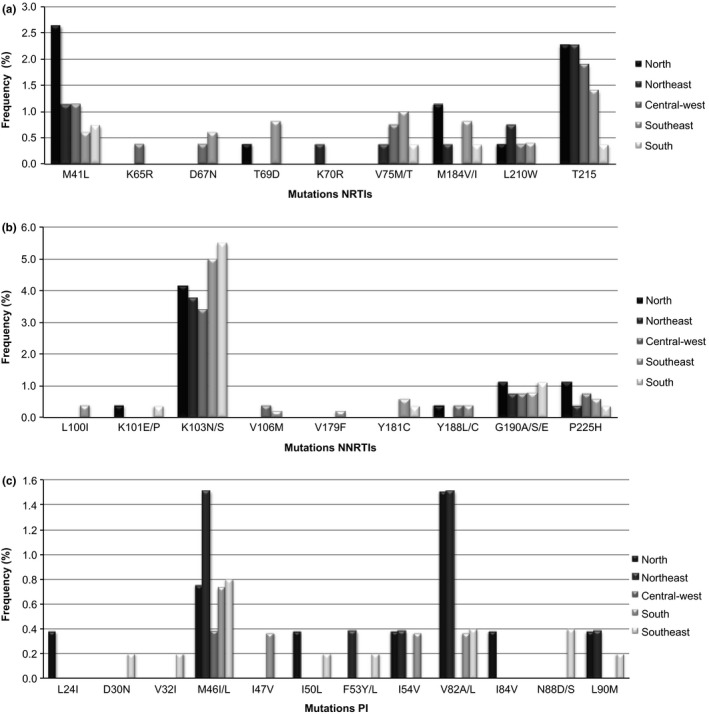
Prevalence of drug resistance mutations by drug class in antiretroviral drug‐naive patients in each of the five geographical regions. (a) nucleoside reverse transcriptase inhibitors (NRTI) (b) non‐nucleoside reverse transcriptase inhibitors (NNRTI) and (c) protease inhibitors (PI).

Concerning the NRTI mutations, M41L was the most prevalent in the north (n = 7, 2.6%) and south (n = 2, 0.7%), whereas T215Y/D/S/E/I/V was highly prevalent in the northeast (n = 6, 2.3%), central‐west (n = 5, 1.9%) and southeast (n = 7, 1.4%). Additional NNRTI mutations were identified at K65, D67, T69, K70, V75, M184 and L210 (Figure 2a).

Regarding NNRTI‐related mutations, K103N/S was the most prevalent variation in all regions: 4.2% in north (n = 11), 3.8% in the northeast (n = 10), 3.4% in the central‐west (n = 9), 5.0% in the southeast (n = 25) and 5.5% in the south (n = 15). The NNRTI mutations were also identified at the positions L100, K101, V106, V179, Y181, Y188, G190 and P225 (Figure 2b).

Regarding the PI mutations, M46I/L was the most prevalent in the north (n = 2, 0.8%), northeast (n = 4, 1.5%), central‐west (n = 1, 0.4%), southeast (n = 4, 0.8%) and south (n = 2, 0.7%). V82A/L mutations were highly prevalent in the northeast (n = 4, 1.5%) and north (n = 4, 1.5%). PI mutations were also detected at L24, D30, V32, M46, I47, I50, F53, I54, V84, N88 and L90 (Figure 2c).

### 3.3 HIV‐1 subtyping assignment

**Figure 3:**
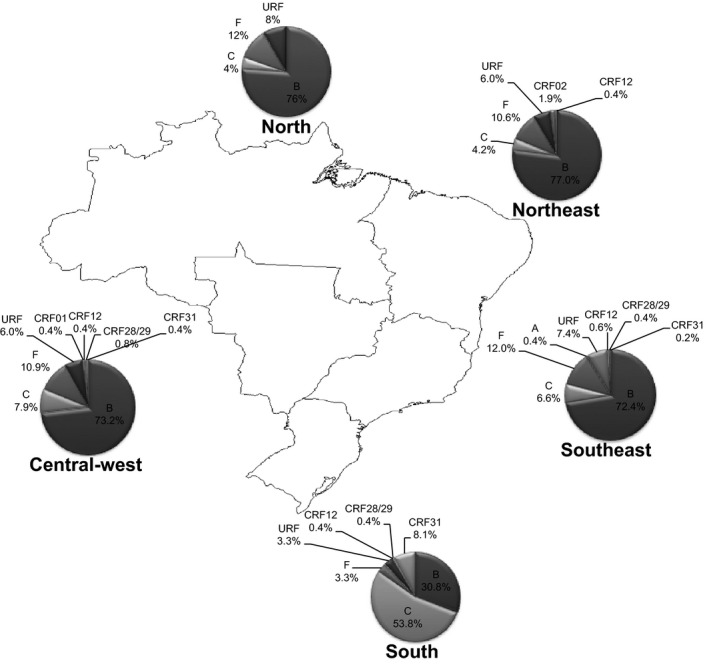
Distribution of subtypes throughout Brazil's five geographical regions. The map shows the distribution of subtype based on PR and RT genomic regions as well CRFs and URFs.

The HIV subtype characterization was evaluated for the 1568 analysed *pol* sequences using the REGA HIV‐1 subtyping tool 19, 20 and HIVdb Program 18. With the exception of the south, the subtype B (n = 1045, 66.8%) remains the most prevalent in Brazil, followed by subtypes C (n = 223, 14.2%) and F (n = 156, 10%). We also found CRFs composed of subtypes B, C and F sequences spread throughout the regions. The CRF31 (B/C) was present in the southern region accounting for 8.1% of all sequences analysed for this region, and the CRF31 variant was also found in the southeast (0.2%) and central‐west (0.4%). In addition, CRF 12 and 29, composed of B and F sequences, were found in the central‐west (0.4% CRF12 and 0.8% CRF29), southeast (0.6% CRF12 and 0.4% CRF29), south (0.4% CRF12 and 0.4% CRF29) and northeast regions, where only CRF12 was found (0.4%). CRF01_AE and CRF02_AG were identified in the central‐west and northeast regions respectively (Figure 3). Unique recombinant forms (URFs) composed by complex subtype pattern between B, C, F and K sequences were found all over the country, accounting for 6.3% of all isolates analysed. No differences between prevalence of TDR among different subtypes have been observed.

Subtype C was the most prevalent in the south of Brazil (n = 147, 53.8%), followed by subtype B (n = 84, 30.8%), CRF31 (n = 22, 8.1%) and subtype F (n = 9, 3.3%) (Figure 3).

## 4 Discussion

This is the first survey including samples from all Brazilian states, therefore truly representative of all five Brazilian geographical macroregions, analysing 1568 samples of recently diagnosed individuals collected between 2013 and 2015. It is interesting to noticed that the majority of our subject included, young male, reflects the new HIV wave of epidemic affecting young MSM (http://unaids.org.br/estatisticas/).

Our data enabled us to demonstrate the prevalence of TDR, varying from 6.8% (n = 18) in the central‐west to 11.2% (n = 56) in the southeast. Based on the WHO HIVDR classification, all Brazilian macroregions showed intermediate level of resistance (5% to 15%). These prevalences are similar to the ones previously described in Brazil, as seen in Figure 1. However, higher prevalence has occasionally been described in the cities of Salvador, located in the northeast region of Brazil (18.9% and 17.0%) 21, 22 and Santos 23, in the southeast region of Brazil (29.2% and 17.0%) 22, 24. Our data is also consistent with other South American countries where mass treatments are provided. In a similar study done in Argentina, researchers found 14% of TDR and 11% of NNRTI DRM 25.

Although this study has not been designed to measure the transmitted resistance in each Brazilian state/city separately, we were able to observe that the state of São Paulo has presented higher levels of SDRM (surveillance drug‐resistance mutation) than the other studied regions (data not shown), reaching 14% (28 resistant samples from 198 analysed). São Paulo, the most populous state in Brazil, was the first to start treatment with antiretrovirals in the 1990s; more than 40% of patients receiving antiretroviral treatment in Brazil are in this state, whereas the city of São Paulo alone is responsible for almost 25% of all antiretroviral treatment in Brazil. Therefore, it is conceivable that long‐term exposure to antiretrovirals, which relates to sequential monotherapy and exposure to unboosted PIs, could increase the odds for antiretroviral failure and consequent TDR.

We observed a high prevalence of K103N – the NNRTI mutation – in recently diagnosed individuals in all regions of Brazil, ranging from 3.4 to 5.5%, compatible to world trends for this drug class 26. There have been speculations that NNRTI mutations can be more readily transmitted due to the higher exposure to this drug class, as well as to its limited effect on the replicative capacity of the virus, therefore persisting for longer periods of time. This is a great concern in Brazil, since the protocol for initial treatment advocates the fixed dose combination of tenofovir/3TC/efavirenz (http://www.aids.gov.br/pcdt) 27. It is also interesting to note that, as seen in Figure 2b, and in accordance to the frequent antiretroviral exposure in Brazil, the efavirenz related NNRTI pathway for resistance 25, which includes mutation at codons 103 associated to codons 100, and 225, is more frequently observed than the nevirapin pathway for resistance, which includes mutation at codons 181 and 101, the latter leading to cross‐resistance to etravirine. Mutation at codon 103 was followed by NRTI mutations, with a high prevalence of the so‐called T215 revertants (215 D/I/V/S, Figure 2a), which are products of the evolution of T215Y or T215F. Although the revertants per se do not present any level of phenotypic resistance 28, it is plausible that individuals harbouring these revertants may also harbour the T215Y or T215F strains, which are associated with virological antiretroviral failure 29. Of course, it is theoretically possible that the revertants were transmitted rather than the original T215Y or T215F strains, and the analysis of minority HIV populations using next‐generation sequencing techniques may be able to answer this important and interesting question. Again, in this study, the mutation at codon 184, generally the more prevalent secondary resistance mutation, has a very low prevalence in this study, possibly related to the higher probability of this mutation to revert to a wild‐type over time 27, which may be a note of caution among individuals presenting TDR mutations. The presence of minority HIV populations presenting M184V/I mutations among individuals already presenting TDR may also be a subject for future studies using next‐generation sequencing techniques in TDR surveys.

It is important to note in Figure 2 panel C that prevalence of PI related TDR is lower than previously documented in Brazil 13, 30, possible related to a trend of increased use of boosted PIs over time which is also associated to a decreased prevalence of secondary PI resistance mutations over time in Brazil 30. Nonetheless, it is also interesting to note that, mutations at protease codons 30, 88, 46 and 82, which relate to PIs such as nelfinavir, indinavir and ritonavir, which have not been used in Brazil for very long, are still being transmitted. This finding suggests that this TDR mutation has been occurring, unnoticed, from patient to patient for a very long time.

In accordance to what has been previously shown in Brazil 31, the subtype C is more prevalent in the southern region, probably due to a founder effect, accounting for prevalence over 50%. Subtype C was also present in all regions of Brazil, in people who were recently diagnosed with HIV‐1 infection, showing its spreading capacity. Together with subtype C, we found a substantial amount of CRF31 isolates in our sampling. This CRF was more present in the southern region; however, we encountered them throughout the central‐west and southeast regions as well. According to predictions using the Bayesian Markov chain Monte Carlo (MCMC) methods and the reversible‐jump MCMC method, HIV‐1 subtype B emerged in 1971, subtype F emerged in 1981, BF recombinants emerged in 1989, subtype C emerged in 1987 and BC recombinants emerged in 1992 32, 33, 34. This study also predicted that the basic reproductive number of secondary infections (*R*_*0*_ = 5 year interval) is 2.4 for Brazilian subtype B strains, 2.3 for subtype F and 4.6 for subtype C, warning for the faster expansion of this latter in the Brazilian epidemics 32. It is also worth mentioning, that one study analysing phenotypic resistance in a limited number of samples from antiretroviral‐naive individuals in Brazil revealed that some subtype C strains presented phenotypic resistance no NRTIs and more frequently to NNRTIs without significant genotypic mutations, which suggests that the genotypic correlates of subtype C resistance might not yet be clearly defined, posing an additional problem related to the HIV‐1 genetic diversity 35.

## 5 Conclusions

In conclusion, we believe that the sampling technique used herein provides, for the first time, results on TDR that are truly representative of Brazil. The results presented reveal a moderate rate of primary prevalence of TDR in the five Brazilian macroregions. These results further illustrate the importance of surveillance studies in the development of future strategies for mitigating TDR or initial treatment‐related strategies, as well as for helping to predict future trends in other regions of the globe, where mass ARV treatment was implemented.

## Competing interests

The authors have no competing interests to declare.

## Authors’ contributions

MBA generated and analysed data. CCC executed statistical analysis, LTB, CMC, CRBA, STSL, HTK, AWA, AOPE, AMSC, MR, JCCF, SBF, ICMO, LEP, HHCW, SBF, MIMCP, MVCF, FMT, RSD and GISLL worked on sample collection and genotyping tests. RBLF organized sampling and reagent distribution logistics and proofread the article. NMCV analysed the data and wrote the article. AFP, MF and FM collaborated on project design, sampling and reagent distribution logistics. AT conceived and designed the study.

All authors have read and approved the final version of the article.

## Funding

This study was supported by funding from the Brazilian Ministry of Health, approved in process number TC 298/12.

## Supporting information


**Table S1.** Distribution of samples stratified in Brazil's five major geographical regions
**Table S2.** Prevalence of drug resistance according to State

## Appendix 1

### Other members of HIV‐BResNet

Andrea de Melo Xavier Shimizu (Fundação Medicina Tropical do Amazonas), Célia Regina Mayoral Pedroso Jorge PhD (Laboratório de Pesquisa ‐ LAPI Universidade Federal da Bahia ‐ Hospital Universitário”Prof. Edgar Santos”), Leda Maria Simões Mello, MSc (LACEN/CE), Eider Gurgel de Freitas (LACEN/DF), Unaí Tupinambás, PhD (Universidade Federal de Minas Gerais ‐ UFMG, Faculdade de Medicina ‐ Laboratório de Imunologia e Biologia Molecular ‐ DIP), Dijane Cristina de Barros Rosa Costa, BSc (LACEN/MS), Sirleide Pereira da Silva, BSc (LACEN/PE), Maria da Graça Winhescki, BSc. (Laboratório Municipal de Curitiba), Carlos Silva de Jesus (Laboratório de AIDS e Imunologia Molecular ‐ Departamento de Imunologia ‐ FIOCRUZ), Érica Ramos dos Santos Nascimento, BSc. (Hospital Universitário Clementino Fraga Filho), Fatima Ercília de Oliveira Prazim, BSc (Instituto de Biologia do Exército), Marilda Tereza Mar da Rosa (LACEN/RS), Karina Salvador, BSc (Laboratório do Hospital Nossa Senhora da Conceição), Senele Ana de Alcântara Belettini, BSc (LACEN/SC), Rejane Maria Tommasini Grotto, PhD (Laboratório de Carga Viral, Hemocentro de Botucatu ‐ UNESP), Paulo Henrique de Oliveira, BSc (Laboratório de Pesquisa em AIDS‐Hospital de Clíncas da UNICAMP), Érica Valessa Ramos Gomes (Laboratório de Biologia Molecular‐Instituto Adolfo Lutz de São José do Rio Preto), Danilo Araujo Dias, BSc and Juliana Galinskas, MSc (Laboratório de Retrovirologia‐UNIFESP, Escola Paulista de Medicina), Norberto Camilo Campos, BSc (Laboratório de Retrovírus. Instituto Adolfo Lutz Central ‐ São Paulo), José Boullosa Alonso Neto (Ministério da Saúde ‐ Secretaria de Vigilância em Saúde ‐ Departamento de Vigilância, Prevenção e Controle das DST, AIDS e Hepatites).
