# Supplementary material for: Brazilian network for HIV Drug Resistance Surveillance (HIV‐BresNet): a survey of treatment‐naive individuals
Source: J Int AIDS Soc. 2018 Mar 5;21(3):e25032. doi: 10.1002/jia2.25032 (PMC5835841; doi:10.1002/jia2.25032)
Supplement: Supplementary file 1 — Table S1. Distribution of samples stratified in Brazil's five major geographical regions Table S2. Prevalence of drug resistance according to State [file JIA2-21-e25032-s001.pdf]

Supplementary material 1: Distribution of samples stratified in the five majors Brazilian geographic regions

Brazilian geographic regions	States	Number of calculated samples	Number of processed samples
NORTH	Acre	4	5
	Amazonas	72	81
	Amapá	9	10
	Pará	111	107
	Rondônia	31	29
	Roraima	10	12
	Tocantins	17	21
Total		254	265
NORTHEAST	Alagoas	13	17
	Bahia	55	62
	Ceará	28	34
	Maranhão	28	31
	Paraíba	16	19
	Pernambuco	71	66
	Piauí	15	7
	Rio Grande do Norte	15	16
	Sergipe	13	13
Total		254	265
CENTRAL-WEST	Distrito Federal	52	60
	Goiás	81	63
	Mato Grosso do Sul	54	62
	Mato Grosso	66	80
Total		253	265
SOUTH	Paraná	55	51
	Rio Grande do Sul	151	173
	Santa Catarina	49	49
Total		255	273
SOUTHEAST	Espírito Santo	15	22
	Minas Gerais	89	89
	Rio de Janeiro	136	191
	São Paulo	263	198
Total		503	500

Supplementary material 2:Prevalence of drug resistance according to State

Brazilian geographic regions	States	Number of Sequences	Number of Sequences with any SDRM (%)
NORTH	Acre	5	0
	Amazonas	81	7(8.5)
	Amapá	10	0
	Pará	107	12(11.6)
	Rondônia	29	4(13.8)
	Roraima	12	1(8.3)
	Tocantins	21	3(14.4)
Total		265	27(10.2)
NORTHEAST	Alagoas	17	0
	Bahia	59	5(8.5)
	Ceará	34	8(23.5)
	Maranhão	32	2(6.2)
	Paraíba	21	1(4.8)
	Pernambuco	66	5(7.6)
	Piauí	7	2(28.6)
	Rio Grande do Norte	16	0
	Sergipe	13	2(15.4)
Total		265	25(9.4)
CENTRAL-WEST	Distrito Federal	60	4(6.7)
	Goiás	63	4(6.3)
	Mato Grosso do Sul	62	4(6.5)
	Mato Grosso	80	6(7.5)
Total		265	18(6.8)
SOUTH	Paraná	51	3(5.9)
	Rio Grande do Sul	173	17(9.8)
	Santa Catarina	49	4(8.2)
Total		273	24(8.8)
SOUTHEAST	Espírito Santo	22	1(4.5)
	Minas Gerais	89	9(10.1)
	Rio de Janeiro	191	18(9.4)
	São Paulo	198	28(14.1)
Total		500	56(11,2)
